# A Comparison of Postmortem Serum and Pericardial Fluid Total Bilirubin Levels With Antemortem Data

**DOI:** 10.7759/cureus.79089

**Published:** 2025-02-16

**Authors:** Shojiro Takasu, Sari Matsumoto, Kana Sakamoto

**Affiliations:** 1 Department of Forensic Medicine, The Jikei University School of Medicine, Tokyo, JPN

**Keywords:** antemortem, biochemical markers, forensic medicine, postmortem, total bilirubin

## Abstract

Introduction: The accuracy of total bilirubin (T-Bil) measurement in postmortem specimens is unclear because most forensic autopsy cases lack recent antemortem biochemical data to serve as references.

Materials and methods: This study compared T-Bil concentrations between antemortem serum values, postmortem serum, and pericardial fluid values in 37 forensic autopsy cases (postmortem interval within 67 hours) and evaluated their efficacy. Moreover, these values were compared between cases with and without liver cirrhosis (LC).

Results: No significant difference was observed between antemortem and postmortem serum T-Bil levels. Although postmortem pericardial fluid T-Bil concentrations correlated with antemortem values, the pericardial fluid concentrations were significantly lower. The median postmortem pericardial fluid to antemortem serum T-Bil ratio was 0.41 ± 0.27. Although antemortem and postmortem serum T-Bil concentrations were significantly higher in the LC group than in the control group, no significant difference was observed in postmortem pericardial fluid T-Bil values.

Conclusions: This article highlights the utility of measuring serum and pericardial fluid T-Bil levels postmortem. To the best of our knowledge, this study is the first to evaluate the postmortem pericardial fluid to antemortem serum T-Bil ratio. Our results showed that this ratio was close to that reported in living patients. Therefore, postmortem pericardial fluid may serve as a significant alternative sampling source in cases where serum is unavailable. However, further evaluation of its diagnostic efficacy in LC is warranted.

## Introduction

Bilirubin (Bil) is a waste product of heme catabolism and is released during the breakdown of senescent erythrocytes. Unconjugated (i.e., indirect) Bil is transported to the liver, where it is conjugated with glucuronic acid (forming direct Bil) and excreted into the bile. Elevation of Bil levels is a significant indicator of certain diseases such as liver dysfunction, biliary obstruction, and hemolysis [1-4]. Clinically, the sum of direct and indirect Bil is reported as the total Bil (T-Bil) parameter [1,2]. The reference value for T-Bil in living patients is defined as 0.3-1 mg/dL [1].

Postmortem evaluations of liver function using serum biochemical markers such as alanine transaminase, aspartate aminotransferase, gamma-glutamyl transferase, and total bilirubin have been reported in several studies [5-8]. Compared to these markers and others, such as alkaline phosphatase and pseudocholinesterase, T-Bil is reportedly more stable, and its serum concentration postmortem is recommended for the evaluation of liver function in autopsy cases [5]. However, the degree to which postmortem serum T-Bil reflects liver function before death is difficult to evaluate because, in most cases, no recent antemortem biochemical data are available.

In certain forensic autopsy cases, an examination is conducted in the emergency department prior to the declaration of death. The resultant blood test findings are likely to reflect the most recent antemortem conditions.

This investigation aimed to compare antemortem serum T-Bil concentrations, derived from emergency department results obtained prior to death, with postmortem T-Bil in serum to assess the accuracy of postmortem T-Bil measurements. Furthermore, postmortem serum is frequently unavailable due to hemolysis. Therefore, other fluids such as pericardial fluid, cerebrospinal fluid, vitreous humor, and urine are frequently used as alternative specimens for forensic purposes. In our institution, besides biochemical analyses of serum, only biochemical analysis of pericardial fluid is regularly conducted. Consequently, we evaluated postmortem T-Bil in pericardial fluid as an alternative specimen to serum. Secondarily, we compared the T-Bil concentration between liver cirrhosis (LC) cases and control cases and evaluated its diagnostic efficacy.

## Materials and methods

Study design and study population

This study was designed as a retrospective, single-center investigation of forensic autopsies conducted at the forensic department of the Jikei University School of Medicine between December 2021 and June 2023. The inclusion criteria were cases that underwent a full autopsy with the following data available: medicolegal autopsy results, T-Bil concentrations in serum and pericardial fluid from postmortem specimens, and biochemical analysis results, including T-Bil, from the emergency room, defined as antemortem blood results. Cases with unavailable postmortem serum and pericardial fluid specimens or missing biochemical analysis data from the emergency room were excluded. The T-Bil values measured in each postmortem specimen were compared with the corresponding antemortem data obtained in the emergency room. Causes of death were classified based on routine autopsy findings.

The study included 37 patients (29 males and eight females, aged 13-87 years) with a postmortem interval (PMI) within 67 hours (14-67 hours). All cases had been in cardiopulmonary arrest upon arrival at the emergency room, where they received cardiopulmonary resuscitation.

The ethics committee of the Jikei University School of Medicine for biochemical research approved this study (permission number 31-073 9572). This study did not receive specific grants from public, commercial, or not-for-profit funding agencies.

Sample collection and laboratory assay

Postmortem pericardial fluid was obtained during autopsy via pericardial incision using a sterile syringe. After measuring the volume, serum was collected from cardiac blood obtained during heart excision in the autopsy. Once collected, the samples were transferred to a laboratory and stored at −80°C for T-Bil measurement. The maximum duration from sample collection to transfer was three days. T-Bil concentrations were measured using an oxidation method with equipment from SRL (Tokyo, Japan).

Statistical analysis

All statistical analyses were conducted using STATA 16 (StataCorp LLC, College Station, Texas), and a p-value < 0.05 was deemed statistically significant across all analyses. Prior to statistical analysis, a normality test (Skewness/Kurtosis test) was performed for each measured value. Given the significant difference, the Wilcoxon matched-pairs signed-rank test was employed to assess differences in concentrations between postmortem and antemortem data. Comparisons between LC cases and other cases were performed using the Mann-Whitney U test. The Spearman rank correlation coefficient was used to evaluate the correlation between the measured values and the difference between the measured values and the PMI. To predict antemortem serum T-Bil, a regression analysis was conducted between antemortem T-Bil and postmortem serum and pericardial fluid T-Bil levels.

## Results

Comparison between antemortem and postmortem T-Bil concentrations

**Figure 1:**
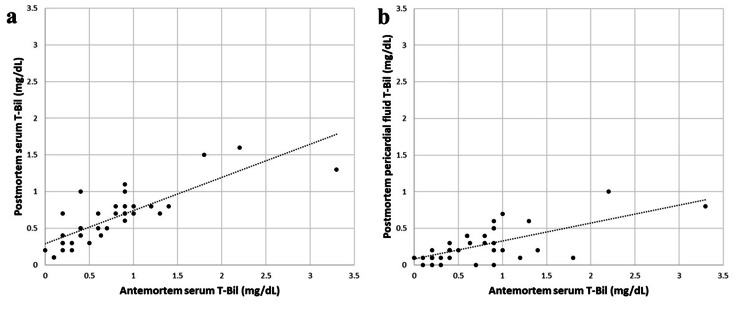
Scatterplot comparing the difference between antemortem and postmortem total bilirubin (T-Bil) concentrations (mg/dL) The dotted line represents the trend line. (a) Scatterplot comparing antemortem and postmortem serum T-bil data (p < 0.05, Spearman’s rank correlation coefficient = 0.83). (b) Scatterplot comparing the antemortem and postmortem pericardial fluid data (p < 0.05, Spearman’s rank correlation coefficient = 0.57).

The sex, PMI, cause of death, and T-Bil concentration of each of the 37 cases are detailed in Table 1. The antemortem serum, postmortem serum, and pericardial fluid T-Bil values were 0.74 ± 0.65 mg/dL, 0.62 ± 0.36 mg/dL, and 0.26 ± 0.24 mg/dL, respectively. No significant difference was observed between antemortem and postmortem serum T-Bil, which returned a Spearman’s correlation coefficient of 0.83 (Figure 1). Although six cases (cases 14, 16, 18, 31, 33, and 36) had higher antemortem serum T-Bil concentrations compared to the reference value in living patients (0.3-1 mg/dL), three of these six fell below the reference value in their postmortem serum samples (i.e., a 50% false-negative rate). One false-positive case (case 7) was observed for the postmortem serum T-Bil level.

**Table 1:** Case details

Case	Sex	Postmortem interval (hours)	Cause of death	Total bilirubin		
				Antemortem (mg/dL)	Postmortem serum (mg/dL)	Postmortem pericardial fluid (mg/dL)
1	M	39	Congestive heart failure	0.9	1	0.6
2	M	28	Ischemic heart disease	0.2	0.3	0.1
3	M	46	Subarachnoid hemorrhage	0.4	1	0.2
4	F	54	Pulmonary embolism	0.2	0.4	0.1
5	M	28	Fat embolism	0.4	0.4	0.2
6	M	42	Ischemic heart disease	0.63	0.4	0.3
7	M	46	Acute heart failure	0.9	1.1	0.5
8	M	24	Acute heart failure	1	0.7	0.2
9	M	36	Ischemic heart disease	0.4	0.5	0.3
10	M	40	Acute myocardial infarction	0.6	0.7	0.4
11	M	43	Subarachnoid hemorrhage	0.3	0.3	0
12	M	36	Ischemic heart disease	0.4	0.5	0.2
13	M	40	Drowning	1	0.8	0.7
14	M	40	Drowning	1.3	0.7	0.6
15	M	41	Hyperthermia	0.8	0.7	0.4
16	M	53	Liver cirrhosis	1.2	0.8	0.1
17	M	42	Pulmonary embolism	0.4	0.5	0.1
18	M	47	Trauma	2.2	1.6	1
19	F	20.5	Asphyxia	0.3	0.2	0.1
20	F	22.5	Gastrointestinal perforation	0.7	0.5	0
21	M	15.5	Acute heart failure	0.1	0.1	0.1
22	F	27.5	Subarachnoid hemorrhage	0.9	0.7	0.5
23	M	29	Urinary tract infection	0.9	0.6	0.3
24	M	38	Anaphylactic shock	0.4	0.4	0.2
25	M	14	Tonsil abscess	0	0.2	0.1
26	M	43.5	Acute myocardial infarction	0.9	0.7	0
27	M	67	Drowning	0.5	0.3	0.2
28	M	33	Acute myocardial infarction	0.6	0.5	0.4
29	M	61	Carbon monoxide poisoning	0.8	0.8	0.3
30	F	36	Diabetes	0.9	0.8	0.2
31	F	30.5	Pernicious anemia	3.3	1.3	0.8
32	M	27.5	Myocardial infarction	0.2	0.2	0
33	M	33	Liver cirrhosis	1.4	0.8	0.2
34	M	29.5	Drug intoxication	0.2	0.7	0.2
35	F	54	Undetermined	0.1	0.1	0
36	F	34.5	Liver cirrhosis	1.8	1.5	0.1
37	M	44	Lung cancer	0.2	0.3	0.1

Although T-Bil concentrations in both postmortem serum and pericardial fluid samples correlated with antemortem values (Spearman’s correlation coefficient, 0.57), the T-Bil concentrations in postmortem pericardial fluid samples were significantly lower than antemortem serum values (Figure 1).

The postmortem pericardial fluid to antemortem serum T-Bil ratio was 0.41 ± 0.27 (after one case was excluded because its antemortem T-Bil was 0 mg/dL). Regression analyses were then conducted between antemortem T-Bil (Y mg/dL) and postmortem pericardial fluid T-Bil (X mg/dL) concentrations. The regression equation for serum T-Bil was Y = 1.80X + 0.27.

Comparison between liver cirrhosis and control cases

**Figure 2:**
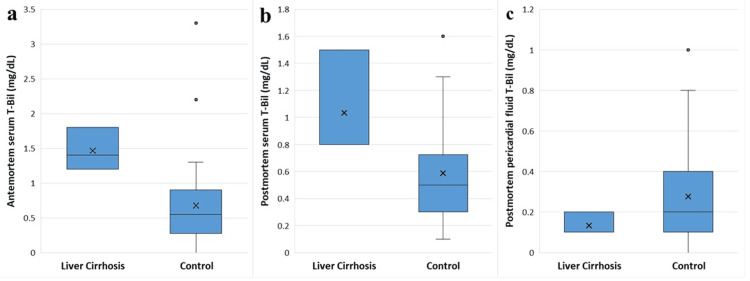
Box chart comparing postmortem T-Bil concentrations (mg/dL) between the cases with liver cirrhosis (n=3) and the control cases (n=34) (a) Antemortem serum. (b) Postmortem serum. (c) Postmortem pericardial fluid. The Wilcoxon matched-pairs signed-rank test was employed to assess the differences in concentrations between postmortem and antemortem data. Antemortem and postmortem serum T-Bil concentrations were significantly higher in the LC group than those in the control group (p = 0.0067 and p = 0.037, respectively). No significant difference was observed in postmortem pericardial fluid T-Bil (p = 0.37).

The antemortem serum T-Bil concentrations in the three cases with LC and the 34 control cases were 1.47 ± 0.31 mg/dL (median, 1.4) and 0.68 ± 0.63 mg/dL (median, 0.55), respectively. The postmortem serum T-Bil concentrations in the LC and control cases were 1.03 ± 0.40 mg/dL (median, 0.80) and 0.59 ± 0.34 mg/dL (median, 0.50), respectively. The postmortem pericardial fluid T-Bil concentrations in the LC and control cases were 0.13 ± 0.06 mg/dL (median, 0.10) and 0.28 ± 0.25 mg/dL (median, 0.20), respectively. Although antemortem and postmortem serum T-Bil concentrations were significantly higher in the LC group than in the control group, no significant difference was observed in postmortem pericardial fluid T-Bil (Figure 2). Moreover, the amount of pericardial fluid did not significantly differ between the LC and control cases.

The amounts of pericardial fluid in the LC and control cases were 6.7 ± 3.5 mL (median, 7) and 22.4 ± 61.9 mL (median, 10), respectively. No significant differences were observed between the groups.

The wide standard deviation in the control group was due to the congestive heart failure case (case 1), which had a pericardial fluid volume of 370 mL.

Relationship between T-Bil and PMI

The differences in antemortem and postmortem serum T-Bil concentrations (postmortem serum concentration - antemortem serum concentration) were correlated with the PMI, but no significant correlation was observed (p = 0.65).

## Discussion

Uemura et al. [5] compared T-Bil concentrations between cases with a PMI of 0-12, 13-24, 25-48, and 49-72 hours. Although a tendency for postmortem elevation was observed, no significant differences were found between the groups. However, 37.3% of the cases showed higher concentrations than the clinical reference values.

Fumeaux et al. [7] compared antemortem with postmortem serum (femoral and cardiac) T-Bil levels in 10 autopsy cases without liver disease (PMI within 48 hours). Their results showed no significant differences between antemortem and postmortem serum T-Bil levels (from both femoral and cardiac blood). Coe et al. [9] reported an increase in Bil with increasing PMI (0.2 mg/dL at 2 hours and 0.7 mg/dL at 20 hours).

In this study, no significant difference was observed between antemortem and postmortem serum T-Bil levels, with a high correlation coefficient of 0.83, leading us to conclude that serum T-Bil levels remain stable postmortem and that postmortem measurement of serum T-Bil levels is useful. Bil is derived from hemoglobin, formed in the monocytic macrophages of the spleen and bone marrow, as well as in hepatic Kupffer cells, and is ultimately released during the breakdown of senescent erythrocytes [4]. Therefore, T-Bil elevation may not occur as a result of postmortem hemolysis. However, previous studies by Uemura et al. [5] and Coe et al. [9] reported postmortem elevation of serum T-Bil levels in vivo; one false-positive case (case 7) was detected in our study. Therefore, the influence of postmortem hemolysis and decomposition on postmortem elevation of serum T-Bil levels cannot be ruled out. However, postmortem values tended to be lower in our study, and 50% of the cases with elevated antemortem T-Bil levels were false positives. Compared to the study by Uemura et al. [5], in which serum was stored for up to a day before analysis, the maximum period from collection to transfer to the laboratory for analysis was three days in our study. Therefore, the possibility of a postmortem decrease in T-Bil levels during the storage period could explain the findings.

Bil levels are elevated in patients with LC due to overproduction of Bil and decreased biliary transport capacity [10]. In this study, significantly higher T-Bil concentrations were observed in LC cases at levels similar to antemortem values, supporting the diagnostic efficacy of postmortem serum T-Bil measurements.

In addition to the LC cases, high T-Bil values were observed in cases 18 and 31. Case 18 was a fatal trauma case with a survival time of 10 days before transportation to the emergency room. Therefore, multiple organ failure that occurred over the agonal period may have contributed to higher T-Bil levels. Case 31 had pernicious anemia, a form of megaloblastic anemia caused by vitamin B12 deficiency [11]. Pernicious anemia causes an elevation in indirect Bil as a result of ineffective erythropoiesis accompanied by intramedullary hemolysis [12].

Postmortem measurements of biochemical markers in blood can be controversial because they undergo postmortem changes. Therefore, fluids such as pericardial fluid, cerebrospinal fluid, vitreous humor, and urine serve as alternative specimens for forensic purposes [7,13-25]. Previous reports have evaluated T-Bil levels in postmortem cerebrospinal fluid, vitreous fluid, and synovial fluid [7,23]. Fumeaux et al. [7] compared postmortem serum and cerebrospinal fluid T-Bil levels in 15 autopsy cases with hepatic steatosis (PMI within 48 hours). Although the measured T-Bil levels were within measurement thresholds, the cerebrospinal fluid-to-postmortem serum ratio varied widely and unpredictably. Naumann et al. [23] measured Bil in vitreous, spinal, and synovial fluids and reported average ratios to serum levels of 220:1, 1:35, and 4:1, respectively.

Pericardial fluid is a transudate of blood, and biomarker concentrations in pericardial fluid reflect those in plasma in living patients [26]. In postmortem pericardial fluid, markers originating from the myocardium may be elevated due to postmortem autolysis, which could render the results unreliable [13-17]. Conversely, our results support the utility of measuring biomarkers that are absent in the myocardium from pericardial fluid instead [18-22]. Although T-Bil could be detected in pericardial fluid, its concentration was significantly lower than that in antemortem serum. Burgess et al. [27] compared serum and pericardial fluid Bil levels in 101 living patients and reported a pericardial fluid-to-serum Bil ratio of 0.44 in pericardial transudate samples.

To the best of our knowledge, no studies have yet evaluated the utility of T-Bil in postmortem pericardial fluid samples. In this study, the postmortem pericardial fluid-to-antemortem serum T-Bil ratio was 0.41 ± 0.27, which was close to the ratio previously reported in living patients [27]. Therefore, our results suggest that T-Bil levels remain stable in pericardial fluid after death. However, we did not find a significant elevation in these levels in our LC cases compared to the control ones. The presence of pericardial fluid related to fluid retention in patients with LC has been reported previously [28]. Therefore, we hypothesized that T-Bil concentrations may have been diluted in our cases due to increased pericardial fluid levels in the LC cases. However, no significant difference in pericardial fluid volume was observed. As the T-Bil concentration was lower in pericardial fluid samples than in serum samples, the sample size may have been insufficient for pericardial fluid to show any significant differences.

This study has several key limitations. The study included only 37 cases; consequently, the evaluation of biomarkers, particularly in relation to the cause of death, was likely insufficient. Moreover, no cases of jaundice, which occurs when serum bilirubin levels exceed 3 mg/dL [29], were included. Although Fumeaux et al. [7] reported no significant difference between postmortem femoral and cardiac sera, Uemura et al. [5] recommended femoral vein blood for the postmortem analysis of T-Bil.

Recent studies have evaluated Bil concentrations in the vitreous humor, cerebrospinal fluid, and synovial fluids and calculated the ratio of the concentration in those fluids to that in serum [7,23]. Consequently, to assess the efficacy of T-Bil concentrations in postmortem specimens, a comparative analysis of samples obtained from multiple sites is required. However, due to the retrospective nature of this study, only data from cardiac blood serum and pericardial fluid were available.

In this study, the possibility of the influence of the storage period on T-Bil concentration cannot be ruled out. To the best of our knowledge, no study has evaluated the effect of storage period on postmortem T-Bil concentration; thus, further studies are required. Other potential confounders, such as undiagnosed liver dysfunction and drugs that can affect T-Bil, were not analyzed in this study. As mentioned before, postmortem evaluation of liver function is difficult because the biochemical markers used in clinical medicine undergo postmortem changes. Hepatotoxic drugs or drugs that inhibit certain enzymes/transporters can cause hyperbilirubinemia [30]. However, in this study, no analysis was conducted on the relationship between T-Bil and drug use due to insufficient prescription data.

## Conclusions

The postmortem measurement of serum and pericardial fluid T-Bil levels is likely valuable for forensic analyses in cases with a PMI of up to 67 hours. Our results demonstrated the utility of postmortem serum T-Bil in identifying liver dysfunction. Moreover, the results indicated that pericardial fluid may serve as an alternative specimen in cases where serum is unavailable. However, further evaluations of the diagnostic efficacy of T-Bil in postmortem pericardial fluid for liver dysfunction and the influence of the storage period on T-Bil concentration remain warranted to ensure the accuracy of postmortem T-Bil measurement.
